# Switching Between LC-ESI-MS/MS and EMIT Methods for Routine TDM of Valproic Acid in Pediatric Patients With Epilepsy: What Clinicians and Researchers Need to Know

**DOI:** 10.3389/fphar.2021.750744

**Published:** 2021-11-23

**Authors:** Ying Xia, Jia-Yi Long, Meng-Yuan Shen, Na Dong, Hong-Li Guo, Ya-Hui Hu, Xiao-Peng Lu, Xuan-Sheng Ding, Feng Chen, Jin-Chun Qiu

**Affiliations:** ^1^ Pharmaceutical Sciences Research Center, Department of Pharmacy, Children’s Hospital of Nanjing Medical University, Nanjing, China; ^2^ School of Basic Medicine and Clinical Pharmacy, China Pharmaceutical University, Nanjing, China; ^3^ Kangda College of Nanjing Medical University, Lianyungang, China; ^4^ Institute of Pharmaceutical Science, China Pharmaceutical University, Nanjing, China; ^5^ Department of Neurology, Children’s Hospital of Nanjing Medical University, Nanjing, China

**Keywords:** valproic acid, LC-ESI-MS/MS, EMIT, switch, TDM, antiseizure medication

## Abstract

**Background:** Valproic acid (VPA) is a widely used antiseizure medication and its dosing needs to be tailored individually through therapeutic drug monitoring (TDM) to avoid or prevent toxicity. Currently, immune-enzymatic assays such as Enzyme Multiplied Immunoassay Technique (EMIT), and Liquid Chromatography (LC)-based techniques, particularly coupled to Electrospray Ionization Tandem Mass Spectrometry (LC–ESI-MS/MS), resulting a potential lack of concordance between laboratories.

**Methods:** In this study, plasma VPA concentrations were determined for 711 pediatric patients with epilepsy by a routine EMIT assay and by a validated in-house LC-ESI-MS/MS method on the same group of samples, aimed to address the aforementioned concern. Consistency between two assays was evaluated using linear regression and Bland-Altman analysis.

**Results:** The calibration curve was linear in the range of 5.00–300 μg/ml for LC-ESI-MS/MS method and 1.00–150 μg/ml for EMIT assay, respectively. The two methods were proven to be accurate with quality control samples. As a result, a significant correlation between two methods was obtained with a regression equation described as [EMIT]=1.214×[LC−ESI−MS/MS]+3.054 (*r* ^2^ = 0.9281). Bland-Altman plot showed a mean bias of 14.5 μg/ml (95% confidence interval (CI) (−0.2, 29.2) and a mean increase of 27.8% (95% CI (3.3, 52.4) measured by EMIT assay more than that measured by LC-ESI-MS/MS method.

**Conclusion:** In conclusion, two methods were closely correlated, but EMIT assay overestimate VPA levels in human plasma compared with LC-ESI-MS/MS method. Due to the observed significant discordance between the tested methods, switching from immunoassays to LC-based techniques for TDM of VPA deserves close attention and therapeutic range of 35.0–75.0 μg/ml may be feasible. However, further studies are needed to evaluate the eligibility of this alternative range in the clinical practice. Clinicians should be informed when switching the VPA quantitation methods during the clinical practice.

## Introduction

Valproic acid (2-propyl-pentanoic acid, VPA), commercially available in most countries during the 1970s, is one of the first-line option for the treatment of epilepsy, especially prescribed in pediatric epilepsy because of its various mechanisms of action and acceptable safety profiles. Additionally, it is being used with increasing frequency for the management of a range of psychiatric conditions (Fleming and Chetty, 2006; Zighetti et al., 2015; Li et al., 2021). Though VPA represents a useful therapeutic alternative in the treatment of epilepsy, it exhibits high inter-subject variability, remarkably when enzyme-inducing or enzyme-inhibiting drugs are co-administered (Methaneethorn, 2018). Also, some adverse drug reactions have been reported including gastrointestinal symptoms, sedation, increased appetite with weight gain, hair loss, tremor, and ataxia (Methaneethorn, 2018; Guo et al., 2019). In addition, approximately only 5–10% of VPA is free in the plasma, and the association between VPA dose and systemic exposure level is curvilinear (Gu et al., 2021). Moreover, a number of factors can exert influence on the VPA protein binding such as age, accompanying medications, renal and hepatic diseases, and pregnancy status, which result in large differences between patients in the plasma concentration-to-dose relationship (Wallenburg et al., 2017; Patsalos et al., 2018). However, a significant association between the decreased seizure frequency and increased serum VPA level was demonstrated (Patsalos et al., 2008). Thus VPA is a good candidate for therapeutic drug monitoring (TDM) to individualize its therapy. Patients with inadequate response, doubtful compliance, intercurrent illness, significant comorbidity, presence of interacting medications and so on can benefit from TDM (Baumann et al., 2004; Patsalos et al., 2008). The recommended VPA therapeutic range for the epilepsy therapy is 50.0–100 μg/ml and the total concentration is usually measured clinically as a reference for treatment (Gu et al., 2021)^,^ (Cook et al., 2016)^.^

The demands for efficient management of many patients with epilepsy have thus advanced the fast, accurate, and precise assays for the antiseizure drug’s monitoring. Gas chromatography (GC)-based methods were the first to be employed for the VPA measurement and played an important role in the clinical studies on VPA (Schobben et al., 1975; Gram et al., 1979). Thereafter, other analytical techniques such as enzyme-multiplied immunoassay technique (EMIT) and fluorescence polarization immunoassay (FPIA), which utilize the same monoclonal antibody against VPA, were widely used (Bowden et al., 1996; Vasudev et al., 2000). They are commercially available, fast, and ease-to-use. However, one potential limitation of EMIT assay for monitoring VPA is fairly low cross-reactivity of certain glucuronide metabolite with antibody used in the immunoassay. In addition, some other disadvantages of the EMIT VPA assay was that the use of EDTA caused a high bias in quantification of VPA (Elyas et al., 1980). High-performance liquid chromatography-tandem mass spectrometry (HPLC-MS/MS) has been used extensively in clinical laboratories over the last 10–15 years (Li et al., 2017). HPLC-MS/MS offers high sensitivity and specificity and is considered to be the gold standard for small-molecule compounds’ analysis. Recently, several HPLC-MS/MS methods for the determination of VPA have been demonstrated (Matsuura et al., 2008; Soni et al., 2016; Wen et al., 2018). They all presented great accuracy and were suitable for routine TDM. However, as we know, the most popular assay to therapeutically monitor VPA in clinical laboratories is still EMIT so far. Up to now, no study is available in literature to compare the analytical results derived from EMIT assay and LC–MS/MS method. The aims of this study were: 1) to develop and validate an LC-ESI-MS/MS method for the analysis of VPA; 2) to evaluate the correlation between EMIT and LC-ESI-MS/MS methods in VPA determination using samples from pediatric patients with epilepsy; and 3) to discuss the method switching from EMIT to LC-ESI-MS/MS for routine TDM of VPA in clinical laboratories.

## Materials and Methods

### Samples

For this study, left-over plasma specimens were tested after completing the VPA assay by EMIT method and reporting results to ordering clinicians. These samples are routinely transported to our lab for monitoring plasma VPA levels in pediatric patients with VPA mono- or poly-therapy. Briefly, 782 blood samples were collected from 711 children with epilepsy (males: 444, females: 267; ranging from 1 month to 18 years, median age: 5 years) at the Department of Neurology, Children’s Hospital of Nanjing Medical University. All samples were collected between March and May 2021. The blood specimens were centrifuged, and the resulting plasma were analyzed immediately for EMIT assay. The left-over plasma samples were separated and stored at −20°C until further LC-ESI-MS/MS analysis. The study was conducted in accordance with the Helsinki Declaration and the study protocol was approved by the Children’s Hospital of Nanjing Medical University ethics committee (Protocol number 202008095-1). This study aimed to assess the analytical concordance of the plasma VPA levels obtained with an EMIT assay and a LC-ESI-MS/MS method, and no clinical and personal data reported. Therefore, the consent to participate is not applicable.

### LC-ESI-MS/MS Method

#### Materials

The reference standard of sodium valproate (purity: 97%; Lot No. 1-MJJ-83-1; expire date: 2024-03-29) and VPA-d6 as the internal standard (IS, purity: 96%; Lot No. 4-LDO-89-3; expire date: 2024-06-04) were taken from the Toronto Research Chemicals Inc (Toronto, Canada). MeOH (Lot No. I1108707035) of HPLC grade was obtained from Merck KGaA (Darmstadt, Germany). Ammonium acetate (NH_4_Ac, ACS Reagent; Lot No. 50Y1905BD) was bought by Sigma-Aldrich, Co. (Wilmington, United States). Ultrapure water was prepared using an in-house Milli-Q water purification system (Millipore, Bedford, MA, United States). Blank human plasma was obtained from the Blood Transfusion Center (Children’s Hospital of Nanjing Medical University, Nanjing, China).

#### HPLC Conditions

Chromatographic separation was performed using a Jasper^TM^ HPLC (AB Sciex Pte. Ltd., Singapore), which is equipped with one SCIEX Dx Controller, SCIEX Dx Sampler, SCIEX Dx Degasser, SCIEX Dx Oven, Jasper HPLC Reservoir, and two SCIEX Dx pumps. A Phenomenex Kinetex^TM^ C18 column (2.1 × 50 mm, 2.6 μm, Torrance, California, United States) and a security Guard-C18 column (4 × 2.0 mm, Phenomenex, Torrance, California, United States) were used for enrichment and separation of VPA and VPA-d6. Gradient elution was designed using a mobile phase consisting of 2 mM NH_4_Ac both in water (phase A) and in MeOH (phase B), at a flow rate of 0.300 ml/min. A gradient program ran through as follows: 0–2.5 min, 40% B; 2.6–3.7 min, 40–95% B; 3.8–5.0 min, 40% B. The column and autosampler were kept at 40 and 4°C, respectively.

#### Mass Spectrometry

The detection was conducted using a Triple Quad™ 4500MD system (AB Sciex Pte. Ltd., Singapore). Quantification was operated with negative ESI multiple reaction monitoring of the following transitions: m/z 143.2→143.1 for VPA and m/z 149.1→149.0 for the IS. Analyst MD software (version 1.6.3, AB Sciex Pte. Ltd., Singapore) was used for the LC-MS/MS system control and data analysis.

#### Preparation of the Calibration Standards and Quality Control Samples

VPA stock solutions (10.0 mg/ml) were prepared in methanol and were further diluted with MeOH: H_2_O (1:1; v/v) to obtain VPA working solutions. All the stock solutions and working solutions were kept at −20°C refrigerator.

Calibration standards and quality control (QC) samples were prepared by spiking appropriate volumes of the working solutions into blank plasma to yield serial concentrations of VPA standard samples. For calibration standards, the concentration levels were 5.00, 10.0, 30.0, 60.0, 120, 200, and 300 μg/ml. The QC samples concentration levels were 5.00 μg/ml (the lower limit of quantification QC, LLOQ QC), 12.0 μg/ml (low QC, LQC), 80.0 μg/ml (medium QC, MQC) and 240 μg/ml (high QC, HQC).

#### Preliminary Experiments

In the study, the left-over plasma samples were not analyzed immediately by LC-ESI-MS/MS method after routine VPA concentration monitoring by the EMIT assay. The way blood samples are processed may have a certain impact on the accuracy of the real concentration of VPA. So, four possible sample handling methods were tested as the preliminary experiments (PEs) shown below for those routine blood samples submitted to our lab.

(PE-a). The routine blood samples were centrifuged immediately for EMIT assay and the whole left-over supernatants were separated and collected. But the plasma samples were stored at −20°C until further analysis.

(PE-b). The routine blood samples were centrifuged and analyzed immediately for EMIT assay. However, the plasma fractions were not separated after centrifugation. Then the whole centrifuged blood samples were stored at −20°C. Before LC-ESI-MS/MS analysis, the blood samples were thawed, and placed for 30 min at bench-top and a 30 μL aliquot of the upper plasma fraction was used for analysis.

(PE-c). The routine blood specimens were treated and stored as method (PE-b) after the EMIT assay. Before LC-ESI-MS/MS analysis, the blood samples were thawed and centrifuged again, then a 30 μL aliquot of the resulting plasma sample was used for monitoring VPA concentration.

(PE-d). The routine blood samples were treated and stored as method (PE-b) after the EMIT assay. Once thawed and centrifuged, the resulting whole supernatants (plasma fractions) were separated completely and vortexed for 5 min, followed by sample preparation as before for LC-EIS-MS/MS determination.

In addition, in order to assess the possible impact of storage of plasma samples on VPA analysis by LC-ESI-MS/MS, a subgroup of 58 samples were analyzed immediately after EMIT assay and again in different days of 4 days’ storage at −20°C.

#### Sample Clean-Up

After routine VPA concentration monitoring by the EMIT assay, the whole left-over supernatants were performed with procedure (PE-a). Before LC-ESI-MS/MS analysis, the plasma samples were thawed and vortexed sufficiently. Then the plasma sample (30 μL) was added to 570 μL of MeOH containing IS (200 ng/ml). The mixture was vortexed for 10 min and then centrifuged for 10 min (4,285 g, 4°C). The supernatant solution (30 μL) was transferred to another clean 1.5 ml Eppendorf tube containing 870 μL of MeOH: H_2_O (1:1; v/v). Then, the resulting mixture was vortexed well for another 3 min and a 5 μL mixture was injected for LC-ESI-MS/MS analysis.

#### Method Validation

The assay was validated according to the Bioanalytical Method Validation Guideline published by the U.S. Food and Drug Administration (FDA, 2018). In brief, the method validation involved in selectivity, linearity, lowest limit of quantitation (LLOQ), recovery, matrix effects, intra- and inter-day accuracy and precision, stability and carryover.

### EMIT Assay

#### Reagents

Emit^®^ 2000 Valproic Acid Calibrators (Lot No. N1; expire date: 2021-10-28) and Emit^®^ 2000 Valproic Acid Assay (Lot No. N2; expire date: 2022-01-01) were supplied by Siemens Healthcare Diagnostic Ltd. (Newark, New Jersey, United States). Controls of VPA (Lot No. 57370; expire date: 2022-05-15) were obtained from Bio-Rad Laboratories, Inc. (Irvine, United States).

#### Assay Performance

The plasma concentration of VPA was assayed using an automated enzyme immunoassay analyzer (SIEMENS, Munich, Germany). The calibration dynamic range of the assay was 1.00–150 μg/mL. A ± 15% deviation of QC samples was accepted to ensure the accuracy and precision of the EMIT method.

Blood samples were centrifuged for 8 min (2,350 g, RT). Afterwards, the resulting supernatant was injected for analysis immediately.

### Statistical Analysis

All data were statistically analyzed using GraphPad Prism v5.01 (GraphPad Software, La Jolla, CA, United States) and Medcalc (Medcalc Software, Ostend, Belgium). Linear regression analysis was performed to estimate the association between the two assays by GraphPad Prism software. Medcalc software was used to draw a Bland-Altman difference plot, which is helpful in demonstrating the relationship between the differences and the magnitude of measurements, showing any systematic bias, and in identifying possible outliers (Li et al., 2017).

## Results

### LC-ESI-MS/MS Method Development and Validation

A sensitive, selective and rapid LC-ESI-MS/MS method was developed and validated for the quantitation of VPA in human plasma. The blank human plasma from six different sources was tested for selectivity and the results proved that no endogenous substances interfered with VPA and IS. The LC-ESI-MS/MS method was linear over the range of 5.00–300 μg/ml and the LLOQ was 5.00 μg/ml for VPA with a signal-to-noise ratio higher than 5. The intra- and inter-day accuracy and precision of the method were all acceptable according to the FDA guidance. No matrix effect or carryover was observed. The full validation data are shown in Supplemental Material.

### LC-ESI-MS/MS PEs

The four different sample handling procedures used for LC-ESI-MS/MS analysis were described in section “*Preliminary Experiments*”. The samples were retested using the four different handing methods, respectively. The deviations of (PE-a) to (PE-d) between initial and repeat measurements are shown in Table 1.

**TABLE 1:** The deviations (Bias%) of procedure (PE-a) to (PE-d) between initial and repeat measurements.

PE-a (bias%)	PE-b (bias%)	PE-c (bias%)	PE-d (bias%)
0.0 (S1)	−26.0 (S6)	−36.2 (S11)	−0.2 (S16)
2.2 (S2)	−34.4 (S7)	15.0 (S12)	−1.4 (S17)
−5.0 (S3)	−11.2 (S8)	−19.5 (S13)	−6.2 (S18)
−2.7 (S4)	−23.2 (S9)	−71.8 (S14)	−7.6 (S19)
−1.7 (S5)	−38.6 (S10)	−19.0 (S15)	2.4 (S20)

As for the experiment evaluating the effect of storage at −20°C, the VPA concentrations measured by LC-ESI-MS/MS method were within the range of 5.00–300 μg/mL. As a result, the deviations between initial and repeated tests ranged from −11.6 to 6.8% and the mean bias was -3.9% among 58 samples.

### EMIT Assay

A calibration curve with a range of 1.00–150 μg/ml was automatically obtained from the Viva-E automatic enzyme immunoassay analyzer. The concentration was calculated by the following formula:

$$A=R_{0}+K\times \frac{1}{1+e^{-a+b\times \ln C}}$$

where *R*_0_ = 2.21833 × 10^2^, *K* = 2.75793 × 10^2^, *a* = −4.19223, and *b* = 0.870019.

The accuracy and precision of QC samples based on three concentration levels were all within the acceptable criteria.

### Comparison of EMIT and LC-ESI-MS/MS

In total, 782 plasma samples were measured by EMIT assay and then by LC-ESI-MS/MS method. Among those, eight samples were below the LLOQ and were excluded from further statistical analysis. Based on the therapeutic range of 50.0–100 μg/ml, the number of plasma samples measured by two methods is summarized in Table 2. VPA concentrations measured by LC-ESI-MS/MS and EMIT were 5.13–126 μg/ml (median 51.8 μg/ml) and 6.00–154 μg/ml (median 66.2 μg/ml), respectively. The median concentration of the plasma VPA determined by EMIT assay was 127.8% of results obtained from LC-ESI-MS/MS method.

**TABLE 2:** The distribution of the plasma VPA concentration data (number/percentage; *n* = 774), measured by both EMIT and LC-ESI-MS/MS methods, in sub-therapeutic, therapeutic, and over-therapeutic reference ranges in relation to clinical efficacy of VPA for epilepsy treatment.

Concentration distribution		EMIT		
		<50.0 μg/ml	In range (50.0–100 μg/ml)	>100 μg/ml
**LC-ESI-MS/MS**	**< 50.0 μg/ml**	149 (19.3%)	192 (24.8%)	0 (0.0%)
	**(50.0–100 μg/ml)**	2 (0.3%)	361 (46.6%)	60 (7.8%)
	**> 100 μg/ml**	0 (0.0%)	0 (0.0%)	10 (1.3%)

This table shows the numbers and percentages of samples which were “concordant under the range” (<50 μg/ml both for EMIT and LC-MS/MS), “concordant within the range” (between 50 and 100 μg/ml for both the methods), “concordant over the range” (>100 μg/ml for both the methods) and the same for the discordant categories.

**FIGURE 1:**
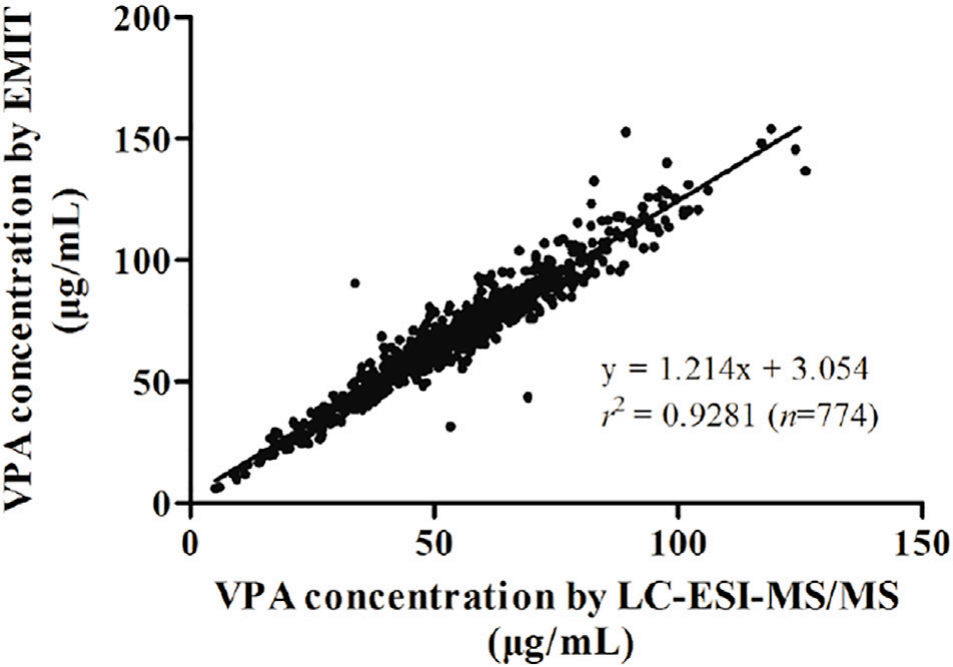
Correlation of the regression curve for LC-ESI-MS/MS and EMIT assay (*n* = 774).

**FIGURE 2:**
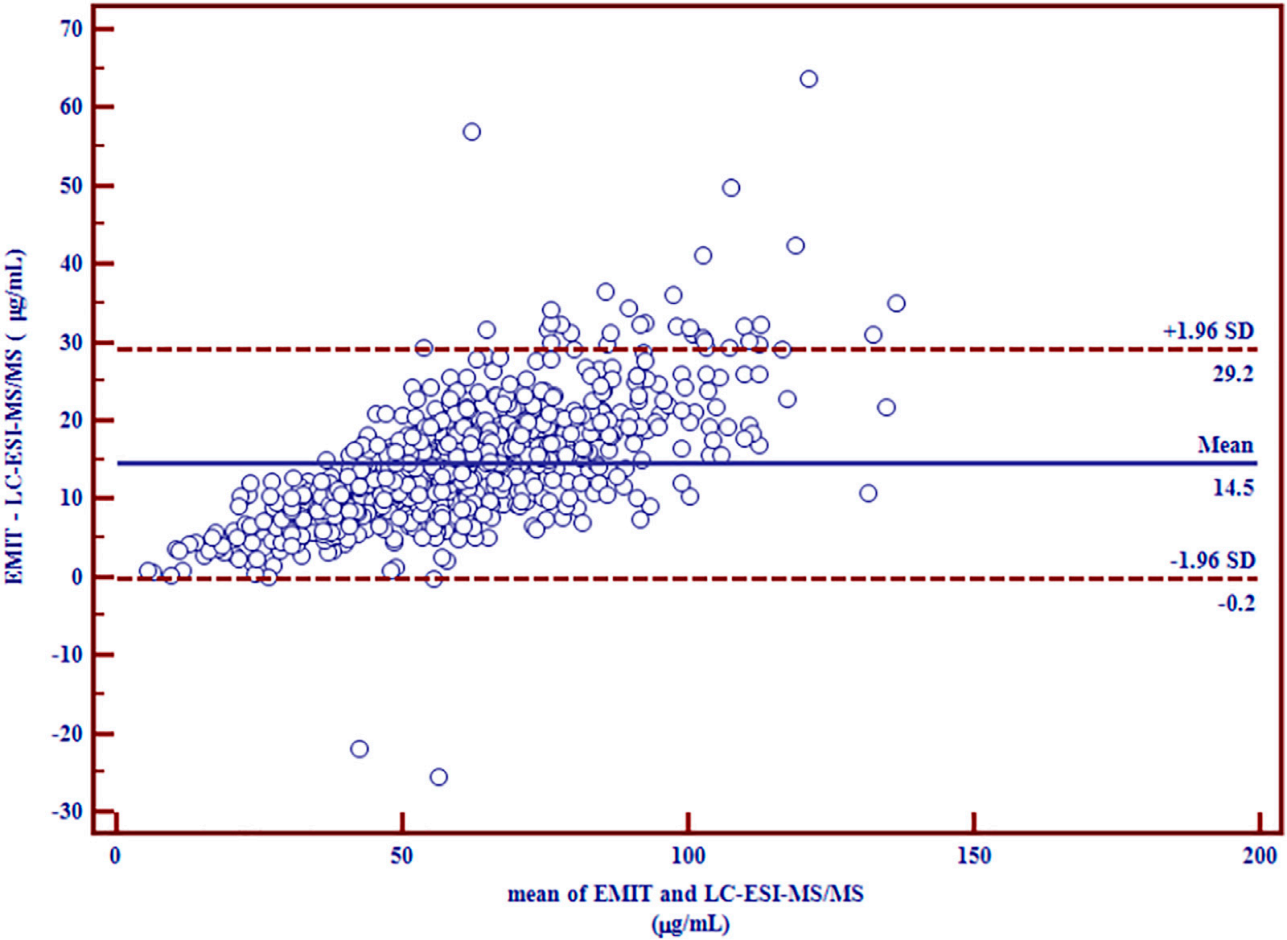
Differences between mean plasma VPA concentrations (µg/ml) measured by LC-ESI-MS/MS and EMIT assay expressed as absolute bias (*n* = 774).

**FIGURE 3:**
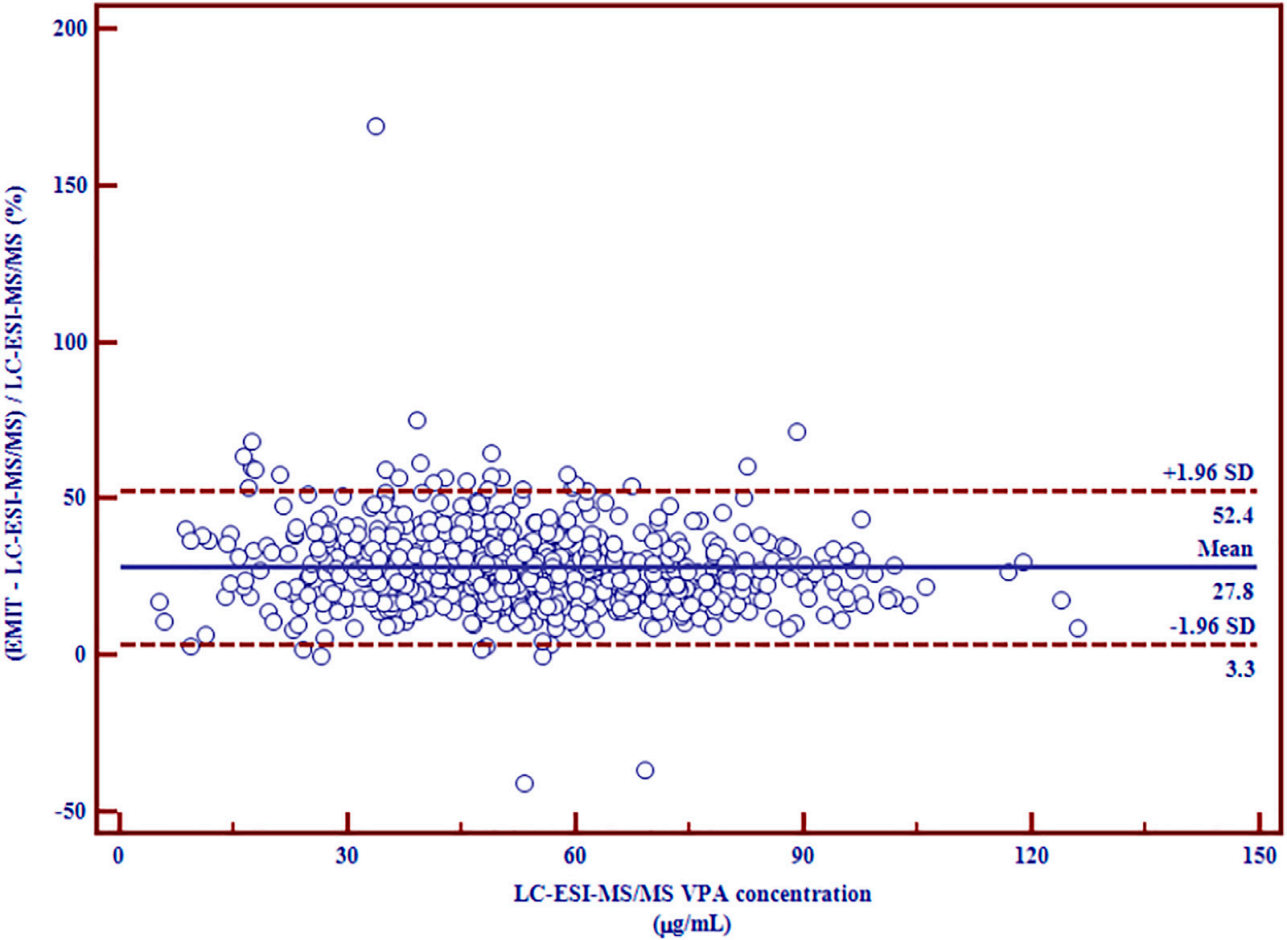
Relative differences between mean plasma VPA concentrations (µg/ml) measured by LC-ESI-MS/MS and EMIT assay expressed in percentage (*n* = 774).

Kolmogorov-Smirnov analysis revealed that the distribution style of the concentration data obtained from LC-ESI-MS/MS or EMIT assay was non-normal distribution. Spearman correlation analysis showed that the data from two methods were significantly correlated (*p*＜0.0001). A regression equation was obtained as following:

[EMIT]=1.214×[LC−ESI−MS/MS]+3.054

with *r* ^2^ = 0.9281 (Figure 1), which indicated a good correlation between the two methods. Nevertheless, the slope was significantly higher than unity (*p* < 0.0001), which reveals the overestimation of EMIT method. The Bland-Altman difference plots of VPA concentration data are presented in Figures 2, 3. Figure 2 shows the disparities between the VPA levels obtained from EMIT and LC-ESI-MS/MS plotted against the mean concentration measured by two methods. As shown in the plots, the concentrations of VPA determined by EMIT assay were higher than those obtained by LC-ESI-MS/MS method (positive bias: 14.5 μg/ml, 95% confidence interval (CI) (−0.2, 29.2). Figure 3 shows the relative difference calculated by [(EMIT)—(LC-ESI-MS/MS)]/(LC-ESI-MS/MS), plotted against the LC-ESI-MS/MS data. EMIT assay overestimation caused a mean relative bias of 27.8% compared with the LC-ESI-MS/MS method and the 95% CI was 3.3–52.4%. In general, both plots demonstrate a systematic overestimation of plasma VPA levels by EMIT with respect to LC-ESI-MS/MS values.

## Discussion

In this study, the left-over plasma samples analyzed by LC-ESI-MS/MS method were performed with four possible sample handling methods described as “2.2.4 Preliminary experiments (PEs)”. Nevertheless, surprisingly, the four different sample handling procedures used for LC-ESI-MS/MS analysis exhibited different accuracy and precision. It was noteworthy that the deviations of method (PE-a) to (PE-d) between initial and repeat measurements were −5.0 to 2.2%, −11.2 to −38.6%, −15.0 to −71.8%, and −7.6 to 2.4%, respectively. Only procedure (PE-a) and (PE-d) could produce repeatable results. It was supposed that the distribution of VPA in the supernatant was not evenly dispersed after freezing, which indicated that the supernatant (plasma fraction) should be vortexed sufficiently once the blood sample had been frozen. Finally, procedure (PE-a) was selected as the standard of practice in this study for LC-ESI-MS/MS analysis.

Moreover, the results of the experiment evaluating the effect of storage shows that LC-ESI-MS/MS method exerted great reproducibility whether the plasma samples were stored at -20°C.

To the best of our knowledge, this is the first study to compare the concentration of VPA measured by LC–ESI-MS/MS and EMIT methods with a large number of samples. The results of this study demonstrated the overestimation by routine EMIT assay compared with LC–ESI-MS/MS, which was in line with previous reports for other medications (Prémaud et al., 2004; Li et al., 2017; Zhou et al., 2020). In the current study, 782 plasma samples from 711 pediatric patients submitted to our lab for routine EMIT assay for VPA monitoring were enrolled. Overall, eight measurements were below the LLOQ and hence were excluded. Finally, 774 concentration data underwent further statistical analysis. A great number of measurements (*n* = 774) enables the reliability of the results. This is one of the major strengths of the current study. As we all know, LC-MS/MS technique has been recognized unanimously to be useful in determination of small molecular chemicals for routine TDM because it is more reliable, selective, and sensitive than EMIT. EMIT technique relies on the reaction between VPA and a biological antibody labeled by glucose-6-phosphate dehydrogenase. The overestimation by EMIT assay could be partly explained by the cross-reactivity of the anti-VPA antibody with other compounds (e.g., glucuronic acid conjugated metabolites, VPA-G). The production insert of Emit^®^ 2000 Valproic Acid Assay shows that “no cross-reactivity” for the EMIT assay based on the testing results for compounds, whose chemical structure would suggest possible cross-reactivity or other therapeutics concurrently used. However, interfering metabolites such as VPAG in the samples were not tested during method validation of the EMIT assay. De Nicolò et al. revealed that the overall comparison between EMIT and LC–MS/MS showing an overestimation by EMIT of 33.5% (De Nicolò et al., 2020). As a result, the disparities between the two methods are noteworthy. In addition, as shown in Table 2, the diagnostic mismatch percentage of VPA concentrations was 32.9% between the two methods, indicating that the results from EMIT and LC-ESI-MS/MS cannot be interchangeable easily. Based on our study, differences in the clinical decision making (diagnostic mismatch) when using EMIT or LC-ESI-MS/MS can be evaluated in the big amount samples (*n* = 774), by comparing results with indication for dose reduction or dose increase by EMIT, but not for LC-ESI-MS/MS. Additionally, clinical laboratory staff should best utilize the same analytical method for routine TDM of VPA, especially for each individual patient. Moreover, clinicians should also be informed when the analytical method has been switched. Timely dose tailoring, if need, should be warranted to avoid drug-related toxicity or loss of antiseizure efficacy.

Potential explanations for the lack of concordance between EMIT assay and LC-ESI-MS/MS for TDM of VPA have been discussed, however, several other factors can also affect TDM activity, such as heterogeneity of each individual sample, drug dosage forms, route of administration, bioavailability, blood sampling time, pathological states, pharmacokinetic interactions, patient compliance and so on. Therefore, standardized operating procedures should be established in clinical practice. Also, consistent detection methods and conditions should be adopted. Furthermore, inter-room quality assessment in clinical laboratories should be conducted regularly to ensure the accuracy and comparability of TDM.

In addition, the early clinical reports published from 1970s to 1990s suggested the therapeutic range of VPA was 50.0–100 μg/ml using GC as the detection method (Schobben et al., 1975; Gram et al., 1979; Henriksen and JOHANNESSEN, 1982; Lundberg et al., 1982). Interestingly, the later literatures used EMIT assay also reported that the therapeutic range of VPA was 50.0–100 μg/ml (Gómez Bellver et al., 1993; Vasudev et al., 2000). In fact, Elyas et al. found that the intercept and higher standard error of the intercept indicated slightly elevated serum concentration of VPA obtained by EMIT in relation to GC, but the concentration difference was acceptable (Elyas et al., 1980). Donniah and Buchanan found that only above or below the therapeutic range (300–700 µM), there was a statistical disparity between the EMIT and GC results (Donniah and Buchanan, 1981), which was line with another report in the same period (Braun et al., 1981). As shown in Table 2, if clinical laboratories would switch the quantitative method from EMIT to LC-ESI-MS/MS, our data suggest that aiming for a lower therapeutic range of VPA (35.0–75.0 μg/ml) may be feasible based on the positive bias of 27.8% measured by EMIT assay compared with LC-ESI-MS/MS.

In addition, the study had potential limitations. VPA is a small molecule, the simple chemical structure of VPA posed challenges for the LC-ESI-MS/MS method. In the study, the parent and daughter ions of VPA and the IS were the same, indicating that no fragmentation was performed and the LC-ESI-MS/MS method was run as pseudo MRM method. As other literatures reported previously (Jain et al., 2007; Matsuura et al., 2008; Soni et al., 2016; Linder et al., 2018; Li et al., 2021), VPA did not produce noticeable fragment ions during ionization. On this basis, it seems that the MS/MS method has the same theoretical selectivity and sensitivity of single-MS spectrometry. In summary, we proved that the use of MRM allowed great sensitivity, accuracy and precision even when employing the same precursor and product ions.

## Conclusion

This is the first study to compare the plasma concentration of VPA measured by routine EMIT assay and thereafter by a novel LC–ESI-MS/MS method using a large number of pediatric blood samples (*n* = 774). In conclusion, EMIT assay overestimated plasma VPA levels by 27.8%, supporting the switch from EMIT to LC–ESI-MS/MS for routine TDM. So far, LC-MS/MS has served as a widespread and efficient technique in many clinical laboratories for monitoring of different medications. Considering the observed significant disparities between EMIT and LC-ESI-MS/MS, switching from immunoassays to LC-based techniques for TDM of VPA deserves close attention and the therapeutic range of 35.0–75.0 μg/ml may be feasible. However, further studies are needed to evaluate the eligibility of this alternative range in the clinical practice.

## Data Availability

The original contributions presented in the study are included in the article/Supplementary Material, further inquiries can be directed to the corresponding authors.

## Abbreviations

CI, Confidence interval; EMIT, Enzyme-multiplied immunoassay; FDA, U.S. Food and Drug Administration; FPIA, Fluorescence polarization immunoassay; GC, Gas chromatography; HQC, High quality control; IS, Internal standard; LC-ESI-MS/MS, Liquid chromatography-electrospray ionization-tandem mass spectrometry; LLOQ, Lowest limit of quantitation; LQC, Low quality control; MQC, Medium quality control; NH_4_Ac, Ammonium acetate; PE, Preliminary experiment; QC, Quality control; TDM, Therapeutic drug monitoring; VPA, Valproic acid.
