# Supplementary material for: Switching Between LC-ESI-MS/MS and EMIT Methods for Routine TDM of Valproic Acid in Pediatric Patients With Epilepsy: What Clinicians and Researchers Need to Know
Source: Front Pharmacol. 2021 Nov 23;12:750744. doi: 10.3389/fphar.2021.750744 (PMC8650496; doi:10.3389/fphar.2021.750744)
Supplement: Supplementary file 1 [file DataSheet1.PDF]

Switching between LC-ESI-MS/MS and EMIT methods for routine TDM of Valproic Acid in Pediatric patients with epilepsy: What Clinicians and Researchers Need to Know

Ying Xia[1,†], Jia-Yi Long[2,†,‡], Meng-Yuan Shen[3,#], Na Dong[4,‡], Hong-Li Guo[1], Ya-Hui Hu[1], Xiao-Peng Lu[5], Xuan-Sheng Ding[2], Feng Chen[1,*], Jin-Chun Qiu[1,*]

[1]Pharmaceutical Sciences Research Center, Department of Pharmacy, Children's Hospital of Nanjing Medical University, Nanjing 210008, China
[2]School of Basic Medicine and Clinical Pharmacy, China Pharmaceutical University, Nanjing 210009, China
[3]Kangda College of Nanjing Medical University, Lianyungang 222000, China
[4]Institute of Pharmaceutical Science, China Pharmaceutical University, Nanjing 210009, China
[5]Department of Neurology, Children's Hospital of Nanjing Medical University, Nanjing 210008, China

[†]These authors contributed equally to this work.
[‡]Visiting graduate student from China Pharmaceutical University
[#]Visiting undergraduate from Nanjing Medical University
[*]Corresponding authors at: Children's Hospital of Nanjing Medical University, 72 Guangzhou Road, Nanjing 210008, China;
E-mail: cy.chen508@gmail.com (Feng Chen) and poheqiu@163.com (Jin-Chun Qiu)

----Supplemental information----

S1 Mass Spectrometry Parameters

Table S1-1 Compound-specific parameters under multiple reaction monitoring mode (MRM)

Compound	Mass transitions (*m/z*)	DP (V)	EP (V)	CE (V)	CXP (V)
VPA	143.2→143.1	-52	-14	-10	-15
VPA-d6	149.1→149.0	-55	-12	-12	-13

Abbreviations: DP, declustering potential; EP, entrance potential; CE, collision energy; CXP, collision cell exit potential.

Table S1-2 Optimized ranges and optimum values of source gas parameters

Parameters	Optimized Ranges	Optimum Values
CAD (Unit)	7 ~ 11	7
CUR (psi)	20 ~ 25	25
GS 1 (psi)	40 ~ 60	45
GS 2 (psi)	40 ~ 60	45
IS (V)	4000 ~ 6000	4500
TEM (°C)	400 ~ 750	550

Abbreviations: CAD, collision gas; CUR, curtain gas; GS 1, nebulizer gas 1; GS 2, nebulizer gas 2; IS, ion spray voltage; TEM, source temperature.

S2 Method Validation Data for HPLC-ESI-MS/MS

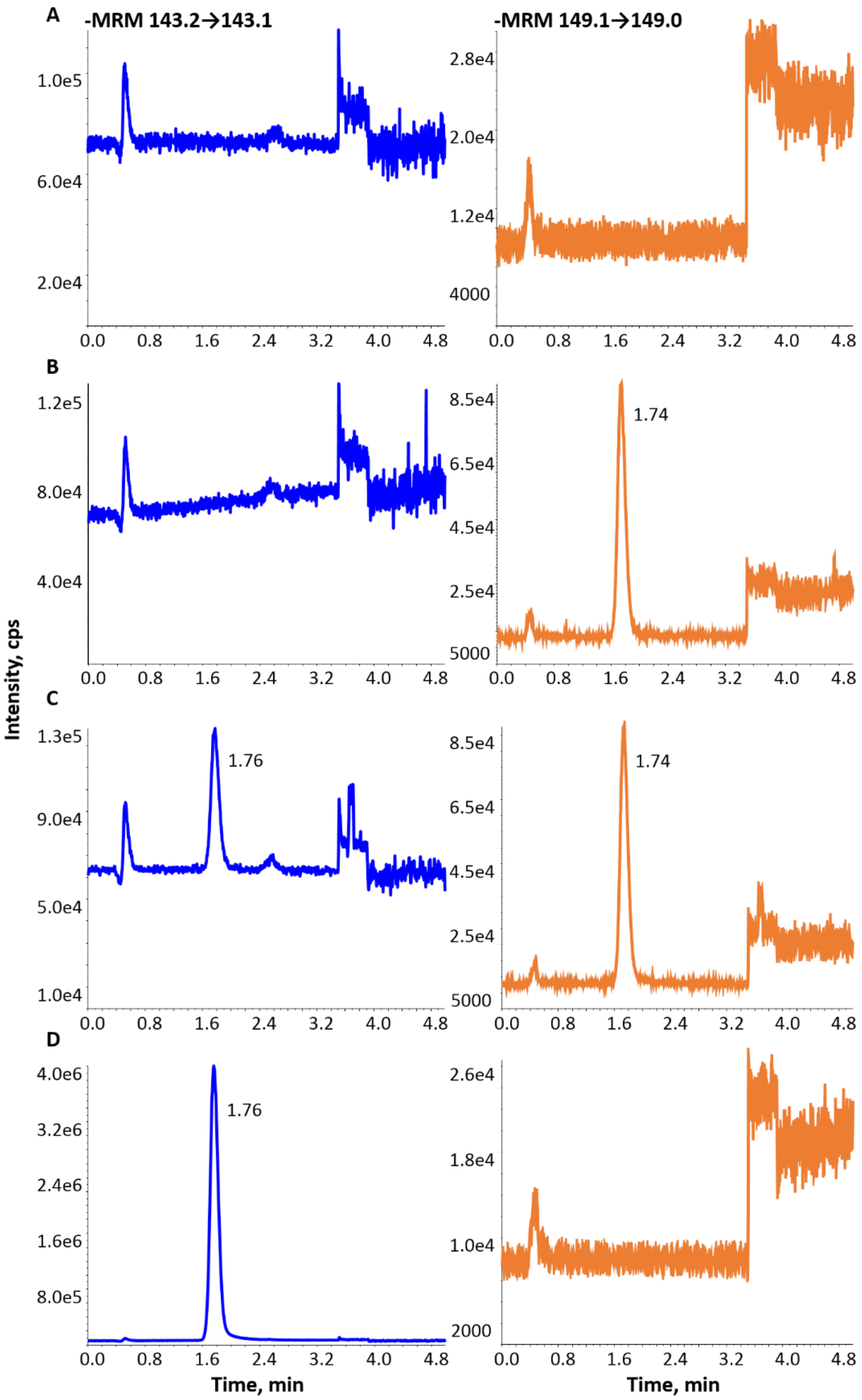

Figure S2-1 Typical multiple reaction monitoring chromatograms of **(A)** double blank sample; **(B)** control blank **(C)** plasma sample spiked with VPA at LLOQ (5.00 μg/mL) and VPA-d6 (IS) at 200 ng/mL; **(D)** ULOQ (300 μg/mL) without IS.

Table S2-1 Precision and accuracy of VPA in human plasma (*n* = 6)

Nominal concentration (μg/mL)	Intra-run (*n* = 6)			Inter-run (*n* = 6 × 3)		
	Measured concentration (mean ± SD, ng/mL)	Accuracy (RE, %)	Precision (RSD, %)	Measured concentration (mean ± SD, ng/mL)	Accuracy (RE, %)	Precision (RSD, %)
5.00	5.47 ± 0.19	9.4	3.5	5.28 ± 0.42	5.6	8
12.0	13.0 ± 0.13	8.3	1	12.2 ± 0.6	1.7	4.9
80.0	76.5 ± 1.3	-4.4	1.7	79.9 ± 7.9	-0.1	9.9
240	251 ± 5	4.6	2	257± 12	7.1	4.7

Abbreviations: SD, standard deviation; RE, relative error; RSD, relative standard deviation; *n*, number of replicates.

Table S2-2 Matrix effect data of VPA in human plasma (*n* = 6)

Nominal concentration (μg/mL)	IS-normalized matrix factor (%)									
	Source of blank matrix						Mean ± SD (%)	RSD (%)	Total Mean (%)	Total RSD (%)
	A	B	C	D	E	F				
12.0	101.5	103.6	103.6	103.9	101.8	105.7	103.4 ± 1.5	1.5		
80.0	103.3	105.4	106.8	105.2	106.0	106.0	105.5 ± 1.2	1.1	103.8	1.5
240	103.5	102.9	102.7	102.9	102.4	99.9	102.4 ± 1.3	1.3		

Abbreviations: SD, standard deviation; RSD, relative standard deviation.

Table S2-3 Recovery data of VPA in human plasma (*n* = 6)

Nominal concentration (μg/mL)		Recovery (Mean ± SD, %)	RSD (%)	Total RSD (%)
	12.0	96.5 ± 2.9	2.8	
VPA	80.0	98.8 ± 2.5	2.5	2.2
	240	94.5 ± 1.7	1.8	
VPA-d6 (IS)	0.200	89.4 ± 1.7	1.9	

Abbreviations: SD, standard deviation; RSD, relative standard deviation.

Table S2-4 Stability of VPA in human plasma (*n* = 3)

Storage conditions	Nominal concentration (μg/mL)	Measured concentration (mean ± SD, ng/mL)	RE (%)	RSD (%)
Room temperature stability (25°C, 12.0 h)	12.0	13.6 ± 0.3	13.3	2.2
	240	257 ± 4	7.1	1.6
Autosampler stability (8 °C, 6 days)	12.0	13.3 ± 0.2	10.8	1.5
	240	248 ± 5	3.3	2.0
Freeze-thaw stability (−20 °C, five cycles)	12.0	12.6 ± 0.6	5.0	4.8
	240	234 ± 5	-2.5	2.1
Long-term stability (−20 °C, 34 days)	12.0	13.5 ± 0.3	12.5	2.2
	240	261 ± 4	8.8	1.5

Abbreviations: SD, standard deviation; RE, relative error; RSD, relative standard deviation; *n*, number of replicates.
